# Psoriatic arthritis is associated with bone loss of the metacarpals

**DOI:** 10.1186/s13075-016-1145-4

**Published:** 2016-10-25

**Authors:** Alexander Pfeil, Laura Krojniak, Diane M. Renz, Lisa Reinhardt, Marcus Franz, Peter Oelzner, Gunter Wolf, Joachim Böttcher

**Affiliations:** 1 Department of Internal Medicine III, Jena University Hospital—Friedrich Schiller University Jena, Erlanger Allee 101, Jena, 07747 Germany; 2 Institute of Diagnostic and Interventional Radiology, Jena University Hospital—Friedrich Schiller University Jena, Erlanger Allee 101, Jena, 07747 Germany; 3 Department of Internal Medicine I, Jena University Hospital—Friedrich Schiller University Jena, Erlanger Allee 101, Jena, 07747 Germany; 4 Institute of Diagnostic and Interventional Radiology, SRH Wald-Klinikum Gera, Straße des Friedens 122, Gera, 07548 Germany

**Keywords:** BoneXpert, Digital X-ray radiogrammetry, Bone mineral density, Psoriatic arthritis, Cortical bone loss, Periarticular demineralisation

## Abstract

**Background:**

BoneXpert (BX) is a newly developed medical device based on digital X-ray radiogrammetry to measure human cortical bone thickness. The aim of this study was to quantify cortical bone loss of the metacarpals in patients with psoriatic arthritis (PsA) and compare these findings with other radiological scoring methods.

**Methods:**

The study includes 104 patients with verified PsA. The BX method was used to measure the Metacarpal Index (MCI) at the metacarpal bones (II–IV). Additionally, the T-score of the MCI (T-score_MCI_) was calculated. Radiographic severity was determined by the Psoriatic Arthritis Ratingen Score (Proliferation Score and Destruction Score) as published by Wassenberg et al. and the Psoriatic Arthritis modified van der Heijde Sharp Score (Joint Space Narrowing Score and Erosion Score).

**Results:**

For the total PsA study cohort, the T-score_MCI_ was significantly reduced by −1.289 ± 1.313 SD. The MCI negatively correlated with the Proliferation Score (*r* = −0.732; *p* < 0.001) and the Destruction Score (*r* = −0.771; *p* < 0.001) of the Psoriatic Arthritis Ratingen Score. Lower coefficients of correlations were observed for the Psoriatic Arthritis modified van der Heijde Sharp Score. In this context, a severity-dependent and PsA-related periarticular demineralisation as measured by the MCI was quantified. The strongest reduction of −30.8 % (*p* < 0.01) was observed for the MCI in the Destruction Score.

**Conclusions:**

The BX MCI score showed periarticular demineralisation and severity-dependent bone loss in patients with PsA. The measurements of the BX technique were able to sensitively differentiate between the different stages of disease manifestation affecting bone integrity and thereby seem to achieve the potential to be a surrogate marker of radiographic progression in PsA.

## Background

Psoriatic arthritis (PsA) is an inflammatory disease characterised by progressive joint destruction and related disability based on enthesitis as well as synovitis, often affecting the small joints of the hand [1]. The radiographic damage in PsA presents a wide spectrum of joint destruction which includes periarticular demineralisation and erosions, joint space narrowing, ankyloses of the joints, malalignment and subluxation of the affected joints and new bony formation with periosteal reaction and ankylosis [2, 3] which are the consequence of the chronic inflammation. Plain radiography has been the gold standard for many decades to assess radiographic damage and progression of PsA and the effectiveness of therapy for individual assessment of each patient and also in clinical trials. In the last decades, scoring methods (e.g. Psoriatic Arthritis Ratingen Score and Psoriatic Arthritis modified van der Heijde Sharp Score (SHS Score)) were established to measure the radiographic damage in PsA [3].

Digital X-ray radiogrammetry (DXR) is a computer-based technique for measuring cortical thickness, and functions as a marker for metacarpal cortical bone mineral density with high precision and reproducibility [4, 5]. A common application of DXR is the quantification of inflammatory-associated periarticular metacarpal bone loss in patients with RA [6]. Periarticular bone loss detected by DXR is strongly associated with disease activity in RA [7] and radiographic progression [8]. Additionally, two initial studies investigated periarticular bone loss in PsA [9, 10].

BoneXpert (BX) is a more advanced DXR technique using computer-assisted diagnosis software for the analysis of the metacarpal bones [11–18]. This new version is now available for the measurement of the Metacarpal Index (MCI) and the quantification of periarticular mineralisation in adults. The clinical advantage of the BX technique consists of its integration into a PACS system as well as into a PACS workflow to reveal a direct image analysis and quantification of periarticular bone loss.

The aim of this study was to evaluate the presence of periarticular cortical bone loss of the metacarpals in patients with PsA using BX and to compare these findings with the established radiological scoring methods. If BX is able to measure bone loss more sensitively it may be considered a surrogate marker of disease progression in the clinical setting.

## Methods

### Study population

A total of 104 PsA patients (57 female, 47 male) fulfilling the CASPAR criteria [19] were included in the study. Radiographs of the hand were performed on all subjects using standardised technical conditions. There was no pre-selection due to severity of PsA or if steroid therapy had been given. All patients were treated either with non-steroidal anti-inflammatory drugs or disease-modifying antirheumatic drugs (details see Table 1).

**Table 1:** Baseline characteristics of the study cohort

	Total study group
Total, *n*	104
Women	57
Men	47
Age (years), mean ± SD	54.7 ± 12.3
Height (cm), mean ± SD	168 ± 9.0
Weight (kg), mean ± SD	79 ± 16.7
Body mass index, mean ± SD	27.6 ± 5.2
Disease duration (years), mean ± SD	9.6 ± 6.7
Tender joint count (0–28 joints), mean ± SD	2.5 ± 2.5
Swollen joint count (0–28 joints), mean ± SD	2.3 ± 2.5
C-reactive protein (mg/l), mean ± SD	9.8 ± 14.2
Erythrocyte sedimentation rate (mm/hour), mean ± SD	16.3 ± 17.1
Corticosteroids, *n* (%)	
Yes (mean dose 5 mg/day)	28 (26.9)
No	76 (73.1)
Non-steroidal anti-inflammatory drug, *n* (%)	49 (47.1)
Synthetic disease-modifying antirheumatic drugs, *n* (%)	42 (40.4)
Biological disease-modifying antirheumatic drugs, *n* (%)	13 (12.5)

*SD* standard deviation

### Measurement of cortical hand bone mass by BX

The BX system (version 2.3.0.4; Visiana, Holte, Denmark) is a computer-assisted diagnostic technique for the radiogeometrical analysis of the metacarpal bones on plain radiographs. All plain radiographs (anterior–posterior projection) of the non-dominant hand were acquired by the same X-ray devices using standardised conditions.

**Fig. 1:**
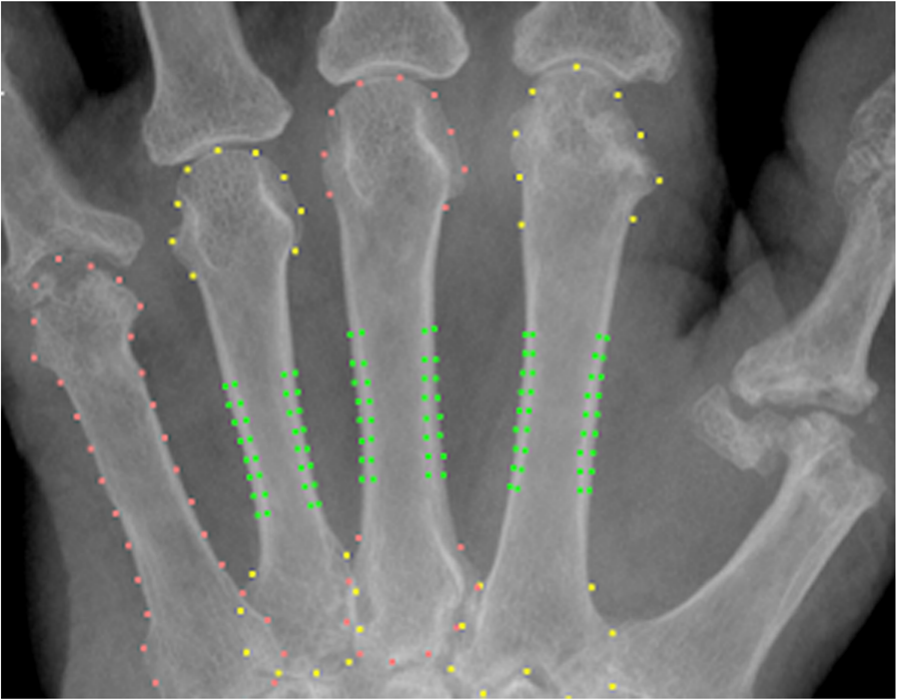
Screen view of BX analysis with the positioned ROI (*green dots*) for the measurement of cortical thickness at metacarpals II–IV

The edge of each metacarpal diaphysis was defined using 32 points which corresponded to the same anatomical locations across all subjects [20, 21]. Two of the points corresponded to the proximal and distal ends of the metacarpal bone, and these markers were used to define the bone axis. The length (L) of the bone was measured along this axis, including the epiphysis. A region of interest (ROI) was positioned at 44 % of L from the proximal end of the bone, and it extended to 25 % of L. The ROIs were located at the metacarpal bones II–IV (see Fig. 1). In this region, the inner and outer edges of the cortical bone partition were determined as follows: the outer cortical edge was the location with the maximal gradient and the inner cortical edge was detected as an intensity maximum [21].

Based on the ROI for each metacarpal bone, the average width (W) and the average cortical thickness (T) were determined and expressed as the mean for the metacarpal bones II–IV in millimetres [21].

The cortical area (A) was estimated by the defined formula for a cylindrically symmetric bone model [21]:

$$ \mathrm{A} = \kern0.5em \uppi \kern0.5em \mathrm{T}\ \mathrm{W}\ \left(1\ \hbox{--}\ \mathrm{T}/\mathrm{W}\right). $$

Additionally, the MCI was computed as the T divided by the W which was later refined to [21]:

$$ \mathrm{M}\mathrm{C}\mathrm{I} = \mathrm{A}\ /\ {\mathrm{W}}^2 $$

Based on the A as well as the metacarpal bone length (L) and W, the Bone Health Index (BHI) was computed as:

$$ \mathrm{B}\mathrm{H}\mathrm{I} = \kern0.5em \mathrm{A}\kern0.5em /\ \left({\mathrm{W}}^{1.333}{\mathrm{L}}^{0.333}\right) $$

### Scoring of hand radiographs

Each radiograph of the PsA cohort was scored by two independent radiologists using the same scoring methods. In the cases of ambiguity, a third highly experienced radiologist reviewed the radiographs for a final decision.

#### Psoriatic Arthritis Ratingen Score

The Destruction Score and the Proliferation Score published by Wassenberg et al. [22] are bicompartment scores used to determine the extent of destructive change (destruction) and the presence of bony growth (proliferation) regarding the joints of the hand and feet in PsA.

The Destruction Score segment indicates the percentage of the joint surface destruction of the articulation with the following scoring: score 0 = normal, score 1 = one or more erosions with destruction of up to 10 %, score 2 = 11–25 %, score 3 = 26–50 %, score 4 = 51–75 % and score 5 = >75 % joint surface destruction.

The Proliferation Score segment evaluates PsA-related bony proliferation using the following grading: score 0 = normal, score 1 = bone proliferation of 1–2 mm or bone growth < 25 % of the original size (diameter), score 2 = bone proliferation 2–3 mm or bone growth with 25–50 %, score 3 = bone proliferation > 3 mm or bone growth > 50 % and score 4 = ankylosis.

The Destruction Score (0–200) and the Proliferation Score (0–160) are added together for a total score of between 0 and 360 points. The individual sum of scoring points is then divided by the number of evaluated joints [22].

#### SHS Score

The Erosion Score segment (total sum points: 280) and the Joint Space Narrowing Score segment (total sum points: 168) [23] of the hand and feet joints were determined using the SHS Score. To assess PsA-specific radiological damage, scores for the distal interphalangeal hand joints and pencil-in-cup/gross osteolysis deformities were added to the original SHS Score as published by Kavanaugh et al. [24]. The SHS Score thus ranged from 0 to 528 (total score sum) which is a composite of the erosion score (0–320) and the joint space narrowing score (0–208) [24]. Additionally, the individual sums of the scoring points were then divided by the number of evaluated joints.

### Statistical analysis

The statistical analysis was performed using SPSS Version 21.0® (SPSS, Chicago, IL, USA), for Windows.

To evaluate reproducibility, 10 measurements of the same radiograph per score were repeated. The results were expressed as mean and standard deviation (SD) and reproducibility errors as a coefficient of variation (CV). The coefficients of variation are typically given on a percentage basis:

$$ \mathrm{C}\mathrm{V}\ \left(\%\right) = \left(\mathrm{standard}\ \mathrm{deviation}/\mathrm{mean}\right)\times 100\ \%. $$

The Pearson coefficient of correlation was used to investigate the association between the BX parameters, age, gender, the Psoriatic Arthritis Ratingen Score as well as the SHS Score.

Thodberg et al. [25] published reference curves for the MCI including healthy European adults. The peak MCI for males is determined at the age of 28 years (MCI 0.6055 ± 0.0509) and for females at the age of 36 years (0.60264 ± 0.0535) [25]. Based on the reference curves and the peak MCI, the T-score_MCI_ of the MCI as a comparative measurement with healthy subjects could be determined. The T-score is calculated as follows [25]:

$$ \mathbf{T}-\mathbf{Scor}{\mathbf{e}}_{\mathrm{MCI}}\kern0.5em =\left({\mathrm{MCI}}_{\mathrm{patient}}\kern0.5em \hbox{--}\ {\mathrm{MCI}}_{\mathrm{peak}}\right)/{\mathrm{SD}}_{\mathrm{peak}} $$

The reduction of all BX parameters for the different scores was assessed using the Mann–Whitney *U* test.

The differences in BX parameters between patients with and without erosions were compared by the Mann–Whitney *U* test.

Sensitivity and specificity of MCI concerning the detection of erosions were based on receiver operating characteristic (ROC) curve analysis. The MCI mean value of the patients with erosions was used as the cut-off value.

The overall significance level was *p* < 0.05.

## Results

### Baseline characteristics

A total of 104 PsA patients (57 women and 47 men) were included in the analysis (see Table 1). The mean disease duration was 9.6 ± 6.7 years. The mean C-reactive protein was 9.8 mg/l and/or the mean erythrocyte sedimentation rate in the first hour was 16.3 mm. In this context, the mean tender joint count was 2.5 ± 2.5 joints and the mean swollen joint count was 2.3 ± 2.5 joints. Forty-nine patients (47.1 %) were treated with non-steroidal anti-inflammatory drugs, 42 patients (40.4 %) received synthetic disease-modifying antirheumatic drugs and 13 patients (12.5 %) received biological disease-modifying antirheumatic drugs. Regarding the use of corticosteroids, 28 patients (26.9 %) were treated with corticosteroids (mean dose 5 mg/day) and 76 patients (73.1 %) received no corticosteroids.

### Reproducibility

The CV was 0 % for BX parameters regarding the Destruction and Proliferation Scores (Psoriatic Arthritis Ratingen Score) and the Erosion and Joint Space Scores (SHS Score).

### BX measurements for the PsA cohort

For the entire PsA study, the T-score of the MCI was −1.289 ± 1.313 SD. Regarding the association of age and BX parameters, a low significant correlation was evaluated (MCI: *r* = −0.585, *p* < 0.001; T-score_MCI_: *r* = −0.586; *p* < 0.001; T: *r* = −0.481; *p* < 0.001). W revealed no significant correlation with age. A significant correlation between the BX parameters and gender was not observed.

### BX measurements compared with standard scoring methods

#### Psoriatic Arthritis Ratingen Score

The BHI, MCI, T-score_MCI_ and T presented significant negative coefficients of correlation with the different scores. The highest negative correlation was observed between the MCI (*r* = −0.771; *p* < 0.001) or the T-score_MCI_ (*r* = −0.775; *p* < 0.001) and the Destruction Score. Similar results were detected between the Proliferation Score for the MCI (*r* = −0.732; *p* < 0.001) versus the T-score_MCI_ (*r* = −0.744; *p* < 0.001). The BHI revealed a significant negative coefficient of correlation (*r* = −0.682; *p* < 0.01) for the Destruction Score and the Proliferation Score. W presented no significant coefficients of correlations to both scores.

#### SHS Score

Lower negative coefficients of correlation were observed between MCI, T-score_MCI_ and BHI and the Joint Space Narrowing Score (MCI: *r* = −0.558, *p* < 0.001; T-score_MCI_: *r* = −0.552, *p* < 0.001; BHI: *r* = 0.522, *p* < 0.01) or the Erosion Score (MCI: *r* = −0.714, *p* < 0.001; T-score_MCI_: *r* = −0.715, *p* < 0.001; BHI: *r* = 0.660, *p* < 0.01). W also showed no significant coefficients of correlation regarding the SHS Score.

### BX results show bone loss depending on severity of PsA

#### Psoriatic Arthritis Ratingen Score

##### Proliferation Score

For the Proliferation Score, MCI (−28.3 %, *p* < 0.01) and T-score_MCI_ significantly differed from 0.596 ± 0.052 (score 0) to 0.427 ± 0.045 (score 4) and from −0.427 ± 0.927 SD (score 0) to −3.616 ± 0.838, respectively (Table 2). The relative difference of T was −31.9 %. In this context, BHI presented a significant difference of −24.6 % (*p* < 0.01) from 5.85 ± 0.42 (score 0) to 4.41 ± 0.52 (score 4). W showed no significant changes between score 0 and score 4.

**Table 2:** BoneXpert parameters in PsA patients stratified by Psoriatic Arthritis Ratingen Score Proliferation and Destruction Scores

Proliferation Score	BHI	MCI	T-score_MCI_	T	W	Destruction Score	BHI	MCI	T-score_MCI_	T	W
0 (*n* = 34)	5.85 (0.42)	0.596 (0.052)	−0.427 (0.927)	2.04 (0.26)	8.02 (1.05)	0 (*n* = 41)	5.88 (0.41)	0.600 (0.053)	−0.346 (0.926)	2.07 (0.25)	8.02 (1.02)
1 (*n* = 28)	5.74 (0.43)	0.576 (0.051)	−0.762 (0.911)	1.99 (0.25)	8.24 (1.05)	1 (*n* = 14)	5.73 (0.39)	0.575 (0.039)	−0.805 (0.710)	1.99 (0.23)	8.23 (1.07)
2 (*n* =17)	5.32 (0.45)	0.531 (0.034)	−1.611 (0.648)	1.81 (0.30)	8.37 (1.13)	2 (*n* =12)	5.44 (0.45)	0.551 (0.026)	−1.281 (0.547)	1.84 (0.29)	8.06 (1.14)
3 (*n* = 14)	5.19 (0.47)	0.505 (0.041)	−2.138 (0.819)	1.73 (0.26)	8.57 (0.84)	3 (*n* = 16)	5.17 (0.44)	0.513 (0.029)	−1.981 (0.627)	1.73 (0.24)	8.32 (0.80)
4 (*n* = 11)	4.41 (0.52)	0.427 (0.045)	−3.616 (0.838)	1.39 (0.23)	8.58 (1.20)	4 (*n* = 13)	5.10 (0.49)	0.494 (0.043)	−2.300 (0.839)	1.72 (0.27)	8.78 (1.03)
–	–	–	–	–	–	5 (*n* = 8)	4.42 (0.75)	0.415 (0.051)	−3.813 (1.035)	1.43 (0.39)	9.04 (1.20)
Absolute and relative changes between score 0 and score 4	−1.44 − 24.6 %	−0.169 − 28.3 %	3.189	−0.65 – 31.9 %	0.56 7.0 %	Absolute and relative changes between score 0 and score 5	−1.46 − 24.8 %	−0.185 *−* 30.8 %	3.467	−0.64 *−* 30.9 %	1.02 12.7 %
Significance	<0.01	<0.01	<0.01	<0.01	NS	Significance	<0.01	<0.01	<0.01	<0.01	NS

Data presented as mean (standard deviation)

*BHI* Bone Health Index, *MCI* Meta*c*arpal Index, *NS* not significant, *PsA* psoriatic arthritis, *T* cortical thickness of the metacarpal bone, *W* width of the metacarpal bone

##### Destruction Score

Using the Destruction Score, BHI presented a significant difference with −24.8 % (*p* < 0.01) from 5.88 ± 0.41 (score 0) to 4.42 ± 0.75 (score 5) (Table 2). MCI and T were also significantly differed between score 0 and score 5 with −30.8 % (*p* < 0.01) and −30.9 % (*p* < 0.01), respectively. The T-score_MCI_ was significantly changed from −0.346 ± 0.926 (score 0) to −3.813 ± 1.035 (score 5). In accordance with the Proliferation Score for W, no significant differences were evaluated.

#### SHS Score

##### Erosion Score

MCI revealed a significant difference of −28.5 % (*p* < 0.01) from 0.601 ± 0.059 (score 0) to 0.430 ± 0.046 (score 5) and T-score_MCI_ showed a significant difference from −0.324 ± 1.039 (score 0) to −3.510 ± 0.912 (score 5) (Table 3). The T and BHI presented comparable differences with −31.3 % (*p* < 0.01) and −24.6 % (*p* < 0.01). W revealed no significant severity dependent change.

**Table 3:** BoneXpert parameters in PsA patients stratified by SHS Erosion and joint space narrowing scores

Erosion Score	BHI	MCI	T-score_MCI_	T	W	Joint Space Narrowing Score	BHI	MCI	T-score_MCI_	T	W
0 (*n* = 33)	5.90 (0.49)	0.601 (0.059)	−0.324 (1.039)	2.08 (0.28)	8.03 (0.95)	0 (*n* = 35)	5.73 (0.57)	0.582 (0.063)	−0.681 (1.163)	1.98 (0.32)	7.99 (0.89)
1 (*n* = 13)	5.80 (0.46)	0.593 (0.042)	−0.554 (0.845)	2.00 (0.28)	7.85 (0.89)	1 (*n* = 26)	5.65 (0.46)	0.568 (0.058)	−0.986 (1.055)	1.96 (0.25)	8.66 (0.94)
2 (*n* =13)	5.59 (0.58)	0.553 (0.055)	−1.154 (1.104)	1.95 (0.33)	8.51 (1.09)	2 (*n* =16)	5.57 (0.43)	0.547 (0.032)	−1.289 (0.591)	1.94 (0.26)	8.19 (1.20)
3 (*n* = 19)	5.28 (0.32)	0.523 (0.028)	−1.800 (0.536)	1.77 (0.21)	8.41 (1.20)	3 (*n* = 15)	5.34 (0.55)	0.537 (0.061)	−1.457 (1.153)	1.82 (0.32)	8.29 (1.16)
4 (*n* = 16)	5.22 (0.36)	0.515 (0.042)	−1.923 (0.754)	1.75 (0.22)	8.46 (1.16)	4 (*n* = 12)	4.52 (0.52)	0.437 (0.050)	−3.416 (0.952)	1.45 (0.23)	8.69 (1.04)
5 (*n* = 10)	4.45 (0.55)	0.430 (0.046)	−3.510 (0.912)	1.43 (0.25)	8.70 (0.95)	–	–	–	–	–	–
Absolute and relative changes between score 0 and score 5	−1.45 − 24.6 %	−0.171 − 28.5 %	−3.186	−0.65 *−* 31.3 %	0.67 8.3 %	Absolute and relative changes between score 0 and score 4	−1.21 − 21.1 %	−0.145 − 24.9 %	−2.735	−0.53 − 26.8 %	0.70 8.8 %
Significance	<0.01	<0.01	<0.01	<0.01	NS	Significance	<0.01	<0.01	<0.01	<0.01	NS

Data presented as mean (standard deviation)

*BHI* Bone Health Index, *MCI* Meta*c*arpal Index, *NS* not significant, *PsA* psoriatic arthritis, *SHS Score* Psoriatic Arthritis modified van der Heijde Sharp Score, *T* cortical thickness of the metacarpal bone, *W* width of the metacarpal bone

##### Joint Space Narrowing Score

The Joint Space Narrowing Score presented a difference of the BHI (−21.1 %, *p* < 0.01) from 5.73 ± 0.57 (score 0) to 4.52 ± 0.52 (score 4) (Table 3). MCI (−24.9 %) and T-score_MCI_ observed a significant difference from 0.582 ± 0.063 (score 0) to 0.437 ± 0.050 (score 4) and from −0.681 ± 1.163 (score 0) to −3.416 ± 0.952 (score 4) respectively. T revealed a significant difference of −26.8 % (*p* < 0.01). Finally, W presented a non-significant difference with 8.8 %.

### BX parameters in dependence on the occurrence of erosions

PsA patients with erosions presented a significantly (*p* < 0.01) lower BHI (−10.2 %), MCI (−12.5 %) and T (−13.9 %) compared with patients without erosion (Table 4). In this context, the T-score_MCI_ in PsA patients with erosions was significant lower (−1.722) than in patients without erosions (−0.324). W (4.4 %; *p* = NS) revealed no significant change between patients with and without erosions.

**Table 4:** Changes of BX parameters dependent on the existence of bone erosions

BX parameter	Without erosions (*n* = 33)	Erosions (*n* = 71)	Difference
BHI mean (SD)	5.90 (0.49)	5.30 (0.60)	−10.2 % (*p* < 0.01)
MCI mean (SD)	0.601 (0.059)	0.526 (0.063)	−12.5 % (*p* < 0.01)
T-score_MCI_ mean (SD)	−0.324 (1.039)	−1.722 (1.190)	1.398 (*p* < 0.01)
T mean (SD)	2.08 (0.28)	1.79 (0.31)	−13.9 % (*p* < 0.01)
W mean (SD)	8.03 (0.95)	8.38 (1.09)	4.4 (*p* = NS)

Data presented as mean (standard deviation)

*BHI* Bone Health Index, *BX* BoneXpert, *MCI* Meta*c*arpal Index, *NS* not significant, *T* cortical thickness of the metacarpal bone, *W* width of the metacarpal bone

The sensitivity and specificity of the MCI regarding the detection of erosions was 88 % versus 49 % (accuracy 81 %, *p* < 0.01).

## Discussion

The BX technique is a recently developed automated method for the measurement of the MCI based on the radiogeometrical analysis of metacarpal bones. The aim of this study was to evaluate the presence of periarticular cortical bone loss of the metacarpal bones in patients with PsA based on the BX method and to compare these findings with different scoring methods.

The MCI is an established measurement for the quantification of metacarpal bone loss, particularly in rheumatoid arthritis [26]. The T-score_MCI_ of the MCI presented a significantly reduced negative value with −1.289 ± 1.313 in all PsA patients. The reduced T-score_MCI_ was clearly associated with a reduced bone mineral density of the metacarpal bones in PsA. Kocijan et al. [27] also found a reduced trabecular bone mineral density of the distal radius and periarticular radius with −12.0 % versus −8.1 % using high-resolution peripheral quantitative computed tomography, in which the bone mineral density was measured proximal to the affected joints.

The comparison of the BX parameters (i.e. MCI) with the SHS Score presented equal coefficients of correlations as reported by Böttcher et al. [8] between the MCI as measured by the X-posure System (the traditional DXR system) and the Sharp Score in patients with rheumatoid arthritis. Focusing on the Proliferation Score and the Destruction Score of the Psoriatic Arthritis Ratingen Score [22], high coefficients of correlation were observed for MCI, precisely reflecting the radiographic changes in PsA by the Proliferation and Destruction Scores.

For all scores, the study found a severity-dependent reduction for the BX parameters (MCI, T-score_MCI_, T and BHI) in PsA patients. The strongest reductions were observed for MCI and T using the Proliferation Score (MCI: −28.3 %; T: −31.9 %) and the Destruction Score (MCI: −30.8 %; T: −30.9 %). The reduced MCI and T is directly associated with cortical thinning and the periarticular demineralisation of the metacarpal bones. Such cortical thinning and periarticular demineralisation show direct association with bone destruction and bone proliferation in PsA. Different cross-sectional studies have reported a strong relationship between reduced metacarpal bone mineral density and MCI as measured by the X-posure System and radiographic joint destruction [8, 26]. For the Sharp Joint Space Narrowing Score and the Sharp Erosion Score a reduced MCI was found (−28.6 % versus −22.1 %) in RA patients [8].

The study also presented a lower MCI (−12.5 %, *p* < 0.01) and cortical thickness as detected by T (−13.9 %, *p* < 0.01) in patients with erosions compared with patients without erosions. These results indicate that the occurrence of erosions is associated with periarticular bone loss. Additionally, the BX technique presented a sensitivity and specificity of 88 % versus 49 % using MCI for the detection of erosions. In this context, the traditional DXR technique showed a sensitivity of 87 % and a specificity of 49 % (for the MCI) in the detection of rheumatoid arthritis [28].

The measurement of periarticular bone loss can be considered a complementary approach to verify PsA-related bony changes and a surrogate marker for PsA progression. The quantification of periarticular demineralisation based on the cortical indices is potentially influenced by the size of the patient. The BHI offers the advantage to quantify cortical thickness and periarticular demineralisation independent of the size of the patient, leading to a better understanding of cortical change.

One limitation of the study is the absence of healthy controls in the BX analysis. The healthy reference cohort data were published by Thodberg et al. [25] and the study used the T-score_MCI_ to quantify the periarticular demineralisation in comparison with healthy subjects. Additionally, a limitation of the study is the absence of longitudinal data regarding therapeutic effects. Hoff et al. [9] presented data about the inhibition of periarticular demineralisation detected by the traditional DXR technique in PsA patients under anti-tumour necrosis factor treatment with infliximab. In this context, the quantification of periarticular demineralisation seems to be a marker for the response of therapy in PsA [9]. However, the data of the study should be used as a basis to compare different treatment strategies in PsA by the BX technique.

## Conclusions

The development of digital imaging and computer-assisted diagnostic methods has enabled a more precise quantification of periarticular demineralisation by the new BX technique. Patients with PsA showed reduced periarticular mineralisation as measured by the MCI and the T-score_MCI_. Additionally, the BX measurements revealed a strong association with the radiographic scoring methods. This new medical device offered the opportunity to quantify severity-dependent periarticular demineralisation in PsA patients with high reproducibility and can function as a surrogate marker of radiographic progression to consecutively optimise an appropriate individual therapeutic strategy.

## Abbreviations

A: Cortical area (mm^2^) estimated by BoneXpert
BHI: Bone Health Index estimated by BoneXpert
BX: BoneXpert
DXR: Digital X-ray radiogrammetry
L: Length of metacarpal bone (mm) measured by BoneXpert
MCI: Metacarpal Index measured by BoneXpert
PsA: Psoriatic arthritis
ROI: Region of interest
SD: Standard deviation
SHS Score: Psoriatic Arthritis modified van der Heijde Sharp Score
T: Cortical thickness of metacarpal bone (mm) measured by BoneXpert
V: Cortical bone volume (mm^2^) estimated by BoneXpert
W: Width of metacarpal bone (mm) measured by BoneXpert

## References

[1] Gladman DD, Antoni C, Mease P, Clegg DO, Nash P (2005). Psoriatic arthritis: epidemiology, clinical features, course and outcome. Ann Rheum Dis.

[2] Day MS, Nam D, Goodman S, Su EP, Figgie M (2012). Psoriatic arthritis. J Am Acad Orthop Surg..

[3] Wassenberg S (2015). Radiographic scoring methods in psoriatic arthritis. Clin Exp Rheumatol..

[4] Böttcher J, Pfeil A, Rosholm A, Malich A, Petrovitch A, Heinrich B, Lehmann G, Mentzel HJ, Hein G, Linss W, Kaiser WA (2005). Influence of image-capturing parameters on digital X-ray radiogrammetry. J Clin Densitom..

[5] Pfeil A, Renz DM, Fröber R, Hansch A, Lehmann G, Sommerfeld J, Malich A, Wolf G, Böttcher J (2015). Influence of angulation on metacarpal bone mineral density measurements using digital X-ray radiogrammetry. Int J Comput Assist Radiol Surg..

[6] Pfeil A, Haugeberg G, Hansch A, Renz DM, Lehmann G, Malich A (2011). The value of digital X-ray radiogrammetry in the assessment of inflammatory bone loss in rheumatoid arthritis. Arthritis Care Research (Hoboken).

[7] Böttcher J, Pfeil A, Mentzel HJ, Kramer A, Schäfer ML, Lehmann G, Eidner T, Petrovitch A, Malich A, Hein G, Kaiser WA (2006). Peripheral bone status in rheumatoid arthritis evaluated by digital X-ray radiogrammetry (DXR) and compared with multi-site quantitative ultrasound (QUS). Calcif Tiss Int..

[8] Böttcher J, Pfeil A, Rosholm A, Petrovitch A, Seidl BE, Malich A, Kramer A, Lehmann G, Hein G, Kaiser WA (2005). Digital X-ray radiogrammetry combined with semi-automated analysis of joint space distances as a new diagnostic approach in rheumatoid arthritis—a cross-sectional and longitudinal study. Arthritis Rheum..

[9] Hoff M, Kavanaugh A, Haugeberg G (2013). Hand bone loss in patients with psoriatic arthritis: posthoc analysis of IMPACT II data comparing infliximab and placebo. J Rheumatol..

[10] Szentpetery A, Heffernan E, Haroon M, Kilbane M, Gallagher P, McKenna MJ, FitzGerald O (2016). Striking difference of periarticular bone density change in early psoriatic arthritis and rheumatoid arthritis following anti-rheumatic treatment as measured by digital X-ray radiogrammetry. Rheumatology..

[11] Thodberg HH, Kreiborg S, Juul A, Pedersen KD (2009). The BoneXpert method for automated determination of skeletal maturity. IEEE Trans Med Imaging..

[12] van Rijn RR, Lequin MH, Thodberg HH (2009). Automatic determination of Greulich and Pyle bone age in healthy Dutch children. Pediatr Radiol..

[13] Martin DD, Deusch D, Schweizer R, Binder G, Thodberg HH, Ranke MB (2009). Clinical application of automated Greulich-Pyle bone age determination in children with short stature. Pediatr Radiol..

[14] Thodberg HH, Jenni OG, Caflisch J, Ranke MB, Martin DD (2009). Prediction of adult height based on automated determination of bone age. J Clin Endocrinol Metab..

[15] Martin DD, Sato K, Sato M, Thodberg HH, Tanaka T (2010). Validation of a new method for automated determination of bone age in Japanese children. Horm Res Paediatr..

[16] Thodberg HH, Neuhof J, Ranke MB, Jenni OG, Martin DD (2010). Validation of bone age methods by their ability to predict adult height. Horm Res Paediatr..

[17] Thodberg HH, Sävendahl L (2010). Validation and reference values of automated bone age determination for four ethnicities. Acad Radiol..

[18] Thodberg HH, Jenni OG, Ranke MB, Martin DD (2012). Standardization of the Tanner-Whitehouse bone age method in the context of automated image analysis. Ann Hum Biol..

[19] Taylor W, Gladman D, Helliwell P, Marchesoni A, Mease P, Mielants H, CASPAR Study Group (2006). Classification criteria for psoriatic arthritis: development of new criteria from a large international study. Arthritis Rheum..

[20] Thodberg HH, Olafsdottir H (2003). Adding curvature to minimum description length shape models. Proc Br Mach Vision Conf..

[21] Thodberg HH, van Rijn RR, Tanaka T, Martin DD, Kreiborg S (2010). A paediatric bone index derived by automated radiogrammetry. Osteoporos Int..

[22] Wassenberg S, Fischer-Kahle V, Herborn G, Rau R (2001). A method to score radiographic change in psoriatic arthritis. Z Rheumatol..

[23] van der Heijde DM, van Leeuwen MA, van Riel PL, Koster AM, van ‘t Hof MA, van Rijswijk MH, van de Putte LB (1992). Biannual radiographic assessments of hands and feet in a three-year prospective followup of patients with early rheumatoid arthritis. Arthritis Rheum.

[24] Kavanaugh A, Ritchlin C, Rahman P, Puig L, Gottlieb AB, Li S, Wang Y, Noonan L, Brodmerkel C, Song M, Mendelsohn AM, McInnes IB (2014). PSUMMIT-1 and 2 Study Groups. Ustekinumab, an anti-IL-12/23 p40 monoclonal antibody, inhibits radiographic progression in patients with active psoriatic arthritis: results of an integrated analysis of radiographic data from the phase 3, multicentre, randomised, double-blind, placebo-controlled PSUMMIT-1 and PSUMMIT-2 trials. Ann Rheum Dis.

[25] Thodberg HH, Böttcher J, Lomholt J, Kreiborg S, Wolf G, Pfeil A (2016). A new implementation of digital X-ray radiogrammetry and reference curves of four indices of cortical bone for healthy European adults. Arch Osteoporos..

[26] Böttcher J, Pfeil A, Rosholm A, Sörös P, Petrovitch A, Schaefer ML, Seidl BE, Malich A, Hansch A, Wolf G, Kaiser WA (2006). Computerized quantification of joint space narrowing and periarticular demineralization in patients with rheumatoid arthritis based on digital X-ray radiogrammetry. Invest Radiol..

[27] Kocijan R, Englbrecht M, Haschka J, Simon D, Kleyer A, Finzel S, Kraus S, Resch H, Muschitz C, Engelke K, Sticherling M, Rech J, Schett G (2015). Quantitative and qualitative changes of bone in psoriasis and psoriatic arthritis patients. J Bone Miner Res..

[28] Pfeil A, Haugeberg G, Renz DM, Reinhardt L, Jung C, Franz M, Wolf G, Böttcher J. Digital X-ray radiogrammetry and its sensitivity and specificity for the identification of rheumatoid arthritis-related cortical hand bone loss. J Bone Miner Meta. 2016. Epub ahead of print. 10.1007/s00774-016-0741-3 26979320

